# Should Skeletal Maturation Be Manipulated for Extra Height Gain?

**DOI:** 10.3389/fendo.2021.812196

**Published:** 2021-12-16

**Authors:** Jan M. Wit

**Affiliations:** Division of Pediatric Endocrinology, Department of Pediatrics, Willem-Alexander Children’s Hospital, Leiden University Medical Center, Leiden, Netherlands

**Keywords:** growth, skeletal maturation, bone age, adult height, aromatase inhibitors, GnRH analogue, growth hormone, predicted adult height

## Abstract

Skeletal maturation can be delayed by reducing the exposure to estrogens, either by halting pubertal development through administering a GnRH analogue (GnRHa), or by blocking the conversion of androgens to estrogens through an aromatase inhibitor (AI). These agents have been investigated in children with growth disorders (off-label), either alone or in combination with recombinant human growth hormone (rhGH). GnRHa is effective in attaining a normal adult height (AH) in the treatment of children with central precocious puberty, but its effect in short children with normal timing of puberty is equivocal. If rhGH-treated children with growth hormone deficiency or those who were born small-for-gestational age are still short at pubertal onset, co-treatment with a GnRHa for 2-3 years increases AH. A similar effect was seen by adding rhGH to GnRHa treatment of children with central precocious puberty with a poor AH prediction and by adding rhGH plus GnRHa to children with congenital adrenal hyperplasia with a poor predicted adult height on conventional treatment with gluco- and mineralocorticoids. In girls with idiopathic short stature and relatively early puberty, rhGH plus GnRHa increases AH. Administration of letrozole to boys with constitutional delay of growth puberty may increase AH, and rhGH plus anastrozole may increase AH in boys with growth hormone deficiency or idiopathic short stature, but the lack of data on attained AH and potential selective loss-of-follow-up in several studies precludes firm conclusions. GnRHas appear to have a good overall safety profile, while for aromatase inhibitors conflicting data have been reported.

## 1 Introduction

There are three therapeutic approaches to increase adult height (AH) for a child with a growth disorder. First, recombinant human growth hormone (rhGH) has been approved for several causes of short stature in children, and in most conditions this treatment (if initiated at a young age) results in an AH within the genetic target height range of the patient. Second, experiments of nature have suggested that keeping the estrogen exposure low in adolescence might delay skeletal maturation and increase AH, which has led to clinical studies on the efficacy and safety of two forms of medication aimed at reducing estrogen exposure, i.e. gonadotropin-releasing hormone (GnRH) analogues (GnRHas) and aromatase inhibitors (AIs). Third, also the combination of rhGH and a GnRHa or AI has been investigated, particularly if the child’s height is still low at onset of puberty. This approach is based on the hypothesis that decreasing estrogen exposure slows skeletal maturation and thereby prolongs the time during which rhGH can stimulate linear growth, and thus results in increased AH. In the present paper I review the available clinical data of the second and third approaches.

Multiple “expert opinions” have been published on this topic [e.g. (1–4) in the last 5 years], including two from our own group (5, 6). The appearance of many reports in the literature describing the effect of GnRHas or AIs in patients with various forms of growth failure, suggests that these compounds are widely used, despite their uncertain efficacy and safety profile, as well as their off-label status for these indications.

One could wonder why one would spend time to produce yet one more “expert opinion” on this topic, while in virtually all classifications of the levels of evidence (7) the expert opinion is considered to be in the lowest grade of scientific value. The answer is threefold. First, I believe that the cumulative observations of published reports (mainly offering a low grade of evidence when assessed separately) on the use of GnRHas for different specific conditions may still lead to general conclusions on their efficacy in growth disorders. This implies that a detailed analysis of strengths and weaknesses of all published data is warranted, followed by a general assessment. Second, it is unlikely that large randomized controlled trials (RCTs) continuing until AH will ever be carried out on these interventions in the various growth conditions, so that I fear that for the next decade the level of evidence will remain as low as it currently is. This implies that it is worthwhile to make an effort to extract as much as possible information from the available reports. Third, I believe that it is important to not only assess the results of formal prospective or retrospective clinical trials, but also observations on “experiments of nature” as well as laboratory data and proxy endpoints that provide supportive evidence that a low estrogen concentration (alone or in combination with a growth promoting agent) is associated with delayed skeletal maturation and increased AH. The complex regulation system of longitudinal growth in the epiphyseal growth plate is influenced by multiple endocrine, paracrine, autocrine and intracrine factors (8), but there is no doubt that the major factor influencing the maturation (“senescence”) of the growth plate is the exposure to circulating and intracellular estrogen (9).

Indeed, there are many clinical observations supporting the concept that AH is dependent of the timing and level of circulating estrogens. Untreated children with central precocious puberty (CPP) reach a considerably decreased AH, which can be normalized by timely treatment with a GnRHa (10, 11). At the other end of the spectrum, individuals with decreased circulating estrogens (for example due to hypogonadism) show an extended period of linear growth, resulting in relatively tall stature, even if they are growth hormone deficient (GHD) (12, 13). Proof that estrogen is the primary factor responsible for closure of the epiphyseal growth plates was delivered by the observation that the growth plates of a man with a defect of the gene encoding the estrogen alpha receptor never closed, resulting in tall adult stature (and osteoporosis) (14). Further confirmation was provided by observations that men with aromatase deficiency demonstrated a similar growth pattern, except that growth could be stopped by administering estrogen medication (15). Additional support of the positive effect of postponing exposure to estrogens is provided by the observation that a late start of estrogen substitution in girls with Turner syndrome increases AH (16), although such delay is generally not advised for psychosocial reasons.

The dose-response relationship between estrogen and short term growth, already investigated several decades ago, shows a biphasic curve, with optimal growth occurring at a low estrogen dose (17–20). This was confirmed by the observation that adding low dose estrogen to rhGH treatment increased growth in young girls with Turner syndrome, although the effect was small (21). At the other side of the spectrum, supraphysiological dosages of exogenous estrogens administered to tall female adolescents inhibit growth of the extremities and decrease AH [but please note that this therapeutic approach has become outdated by potential negative long-term effects on fertility (22, 23)]. A consequence of the apparent biphasic dose-response curve is that complete suppression of estrogen exposure may be less beneficial for growth than partial suppression. A recent study showed indeed that a threshold level of estrogen of 10 pmol/L appeared to be needed for an optimal growth rate corresponding to a normal male pubertal growth spurt (24). I shall come back to this issue in paragraph 3, where the use of AIs is discussed.

For each of the referred papers on efficacy reviewed in this paper, I graded the level of evidence (LoE) of studies in a similar fashion as described by the International Consortium on the use of Gonadotropin-Releasing Hormone Analogs in Children (11) (from now on referred to as “International Consortium”) using 5 levels: LoE 1 (homogenous randomized controlled trials), 2 (meta-analyses or heterogeneous prospective trials), 3 (case-control studies and retrospective cohorts), 4 (uncontrolled cohort and case studies) or 5 (expert opinions, case reports, and personal observations) (7).

## 2 The Effect and Safety of a GnRH Analogue in Children With a Low Predicted Adult Height

On the use of GnRHas in pediatrics, two international conferences have been held. The first was convened in 2007 by the Pediatric Endocrine Society (PES) from the USA and the European Society for Pediatric Endocrinology (ESPE). The US Public Health grading system (25) was used to grade the evidence and strength of the recommendations, and the participants aimed to adhere to modified appraisal of guidelines research and evaluation (AGREE) criteria (26). The resulting Consensus Statement stated that the efficacy of GnRHas for increasing AH in conditions other than CPP “requires additional investigation and cannot be suggested routinely” (10).

Recently, a second conference was organized by a large number of pediatric endocrine societies (International Consortium) (11), which also included a large section on the use of GnRHas in adolescents with gender dysphoria. The participants aimed to “concisely address topics related to changes in GnRHa usage during childhood and adolescence since the previous consensus statement” and the authors stated that the resulting publication “is not a consensus statement and hence has not been endorsed by any of the societies that designated participating authors.” For the purpose of the present review paper, the section on “use of GnRHa in other conditions” is most relevant, and will be discussed in paragraphs 2.2.1 to 2.2.3.

### 2.1 The Effect of a GnRH Analogue Alone

Treatment with a GnRHa as single treatment is usually effective for children with CPP to reach a normal AH (10, 11). The positive results of GnRHa treatment in CPP triggered several investigators to study the effect of GnRHa on AH in short children with relatively early puberty, albeit still within the population reference range. There are two reports of a small positive effect of a GnRHa in such cases. The only placebo-controlled RCT on the effect of a GnRHa on AH was carried out in 24 children with idiopathic short stature (ISS) and 26 with many different diagnoses. Fourteen of them also received rhGH. The authors reported that GnRHa treatment markedly slowed down further BA progression and significantly increased AH by a mean of 0.6 SD, as compared with PAH at baseline. This effect was independent of sex, the presence or absence of concomitant rhGH, and the presence or absence of a growth-limiting syndrome (27) (LoE 1).

A similar effect was suggested in a recent retrospective cohort study, but only for females (28) (LoE 3). In contrast, four other studies (29–32) (LoE 3) showed no effect. As discussed previously (6), and in line with the International Consortium’s opinion (11), my opinion remains that there is insufficient evidence that GnRHa alone increases AH, except for children with CPP.

In order to compensate for the very low height velocity that is often observed during GnRHa treatment in patients with CPP, three approaches have been investigated. First, the combination of GnRHa plus a mini-dose of estrogen was compared with GnRHa alone in patients with CPP in a two year pilot study (33). The results suggested that the low height velocity on GnRHa can be prevented by low-dose estrogen without undue acceleration of bone age (LoE 2), but long-term results have not been reported. Second, the combination of a GnRHa with an anabolic steroid was investigated in two studies with an apparently positive effect on AH compared with matched controls (34, 35) (LoE 3), but since then no more data have been presented. Third, rhGH can be added, which will be discussed in paragraph 2.2.5

There are a number of growth disorders in which predicted adult height (PAH) can be low on conventional disease-specific treatment, which motivated studies on the value of adding a GnRHa. Results of this strategy are discussed in the following paragraphs.

#### 2.1.1 Hypothyroidism

In a retrospective chart review, height outcome and body mass index (BMI) were analyzed in children with severe longstanding hypothyroidism and bone age (BA) delay treated with LT4 alone or with LT4 plus GnRHa (36). Six GnRHa-treated patients and seventeen controls were followed to AH. At diagnosis, GnRHa-treated patients were older and shorter for chronological age, and more advanced in puberty and BA compared to controls on LT4 alone. Despite these baseline differences (which would predict a lower AH for the experimental group), both groups showed similar improvements in height standard deviation score (SDS), similar height deficits and comparable AH, which was interpreted as a positive therapeutic effect (LoE 3). Changes in BMI SDS were similar for both groups (36).

#### 2.1.2 Laron Syndrome

Children with GH insensitivity syndrome (Laron syndrome) treated with recombinant human IGF-I show a modest growth acceleration, but AH is usually still below the population range. A few reports [e.g (37, 38)] suggested that adding a GnRHa may increase AH if started at the onset of puberty (LoE 4), but controlled studies have not been reported.

### 2.2 The Effect of a GnRH Analogue in Combination With Recombinant Human Growth Hormone

Information on efficacy and safety of GnRHa plus rhGH is available for the following conditions: 1) growth hormone deficiency (GHD); 2) short stature after being born small for gestational age (SGA); 3) ISS; 4) haploinsufficiency of *SHOX*, *NPR2* or *ACAN*; 5) CPP; 6) congenital adrenal hyperplasia (CAH); and 7) hypothyroidism. For the first two conditions and *SHOX* haploinsufficiency rhGH treatment is approved in most parts of the world, and for ISS it is registered in the USA and some other countries. For all other conditions reviewed here, the use of rhGH is off label. GnRHa treatment is off-label for all pediatric conditions except CPP.

#### 2.2.1 GH-Deficient Children

The most logical group of GH deficient children who may benefit from the addition of a GnRHa are children who develop CPP before or during rhGH treatment, for example children who had undergone treatment of malignancies (39–42) (LoE 2). The recent conference report stated that adding a GnRHa leads to increased PAH and AH in such cases (11).

A second class of GH deficient children in whom co-medication with a GnRHa can be considered are those who have not reached full catch-up growth at the onset of puberty (11). Longitudinal data on height in rhGH-treated children with GHD have shown that height SDS at the onset of puberty is similar to AH SDS (43, 44). This implies that a low height SDS at pubertal onset, even if this is normally timed, likely leads to a low AH SDS. This was the reason that a prospective RCT was carried out in Santiago (Chile) in collaboration with the National Institutes of Health (Bethesda, USA) to compare near-adult height (NAH) of treatment-naïve GHD patients (in Tanner stage II-III, females premenarcheal) treated with either rhGH plus GnRHa (experimental group, n=7) or rhGH alone (controls, n=10) (45). rhGH was administered until patients reached NAH, and the GnRHa was given for 3 years. BA advancement was significantly different between groups in the 3 year interval (1.5 ± 0.2 “years” on rhGH plus GnRHa versus 4.2 ± 0.5 on rhGH alone), and NAH was-1.3 ± 0.5 versus -2.7 ± 0.3 SDS, respectively (LoE 1). The difference in NAH was close to 10 cm. While I agree with the authors’ conclusion that these results indicate that delaying puberty with a GnRHa in GHD children during treatment with rhGH increases AH, I wish to call the readers’ attention to the fact that the patients in this study were quite different from the usual GHD patient in countries where rhGH is approved and reimbursed for this indication. These severely GH-deficient patients entered into the study at a remarkably late mean age of 14.3 years, BA of 11.3 years, height of -4.3 SDS, and PAH of -3.1 SDS. They are therefore not representative for the majority of GHD patients in whom rhGH is typically started within the first decade of life with less severe growth delay.

One year later our group reported on a retrospective analysis of the effect of the addition of GnRHa (started shortly after the onset of puberty) to rhGH treatment in children with GHD and a low height SDS [mean (SD) -3.0 (1.5)] at onset of puberty (43). Matched controls with rhGH treatment only were used for comparison. The children were younger (mean age 8.9 years), equally short at start of rhGH treatment, but less short at the start of GnRHa compared with the subjects in the Chilean RCT (45). The effect of GnRHa addition on AH [in terms of AH minus target height (TH) SDS] was estimated at 1.2 SDS (approximately 8.5 cm) (LoE 3), so similar to the results of the RCT. We later described an even more impressive height gain of 25-30 cm as a result of the addition of a GnRHa in two siblings with severe GHD due to a homozygous *GHRHR* defect who moved to the Netherlands at mid-puberty and started rhGH plus GnRHa treatment at that time (46) (LoE 4).

In the International Consortium’s report it was concluded that “the addition of a GnRHa to GH at the onset of puberty and treatment for at least 2 years resulted in gains of AH ranging from 6 to 9 cm (∼1–1.5 SD)”, in line with my conclusion. This observation is also compatible with the observations in patients with undiagnosed or untreated combined deficiency of GH and gonadotropic hormones, who can reach a normal height or even turn “from dwarfs to giants” (12, 13).

Regarding safety of this approach, there is a theoretical risk of decreased bone mineral content (BMC) of 2-3 years of GnRHa. This was investigated in the Chilean RCT (45) and in fact patients treated with rhGH plus GnRHa had a significantly lower BMC after 3 years of therapy. This difference, however, did not persist after both groups of patients reached NAH (47). Another potential adverse event is disproportionate growth (potentially leading to a eunuchoid shape), but this was not observed in the RCT (45). The psychological profiles of the patients who participated in this study showed that their first priority was to increase linear growth, whereas pubertal progression was felt to be of lesser importance, particularly for boys (45).

#### 2.2.2 Short Children Born Small for Gestational Age

Pubertal height gain is often less than expected in children born SGA, as a result of an earlier onset of puberty, an earlier peak height velocity, and accelerated bone maturation (11, 48, 49). Therefore, theoretically, it would make sense to add a GnRHa if height SDS is low at pubertal onset in order to increase AH. The results of three studies are in favor of this hypothesis [reviewed in (50)].

First, in a clinical trial on rhGH-treated short early pubertal children born SGA, patients were randomized into 2 groups (rhGH dosage of 1 or 2 mg/m^2^.day). Children with a height below 140 cm (-2.5 SDS in the Netherlands) at pubertal onset received GnRHa co-treatment for 2 years, and their growth was compared with that of children with a height above 140 cm at pubertal onset receiving rhGH only. In children treated with rhGH plus GnRHa, the total height gain on GH treatment in both dosage groups (34.5 cm and girls 24.2 cm for boys and girls, respectively) was larger than expected for the general population, in spite of a shorter pubertal duration after discontinuation of GnRHa (51, 52) (LoE 2). As a result, AH of these rhGH plus GnRHa treated children was similar to AH of rhGH-treated children who were >140 cm at pubertal onset, which would not have been expected without GnRHa co-medication. Extensive studies on this cohort during and up to 5 years after cessation of rhGH regarding body composition, metabolic profile, bone health, cognition, self-perception, behavior and Health-related Quality of Life (HRQoL) showed no adverse effects of the addition of the GnRH analogue (53–57). It is noteworthy that the initially proposed research design (an RCT on the addition of GnRHa) was turned down by the medical ethics committee, because it was expected that adding a GnRHa would have a positive effect on AH.

Second, the combination of rhGH plus GnRHa versus untreated controls for 3 years in short children born SGA or with a normal birth weight (idiopathic short stature, ISS) showed a positive effect on AH in an RCT, but only in girls (58) (LoE 1). Third, a French retrospective study (59) showed that after 4.6 ± 2.8 years of rhGH treatment, height SDS of short children born SGA increased from -2.2 ± 0.9 to -1.5 ± 0.9, and that in a multivariate analysis, the use of a GnRHa therapy for at least 2 years was one of the eight predictive factors (estimated effect 0.4 SD) (59) (LoE 3). The only report suggesting that adding a GnRHa to rhGH treatment had no positive effect on AH was based on a retrospective analysis (60) of non-standardized GnRHa treatment in 16 of 37 patients with Silver-Russell syndrome (LoE 4) (61).

My interpretation of the available data goes a step further than the one taken by the International Consortium (“it is appropriate to consider the potential advantages and disadvantages of treatment with GH and GnRHa in this population”) and contrasts with a recent opposite view (62). I believe that there is sufficient evidence that co-treatment with a GnRHa for 2-3 years does increase AH in rhGH treated short SGA-born children if height SDS is <-2.5 at pubertal onset.

#### 2.2.3 Idiopathic Short Stature

Unlike the previous two conditions, ISS is not an approved indication for rhGH in most parts of the world (except the USA and a few other countries), although reports on post-marketing databases suggest that it has been widely prescribed off label in other countries as well (63). Several studies have been performed on the potential efficacy of the combination of rhGH plus GnRHa in ISS, which can be divided into five sets.

First, there are four reports without follow-up till AH, in which the change in PAH was used as outcome marker for efficacy (64–67). These reports will not further be discussed due to the unavailability of attained AH. The second set contains two uncontrolled studies (68, 69). The third set comprises two controlled studies on a short course of rhGH plus GnRHa (58, 70). Set 4 contains two studies comparing the effect of the combination treatment with that of rhGH alone (71, 72). Set 5 is the retrospective Israeli study in prepubertal and pubertal children on the effect of a GnRHa in addition to long-term rhGH treatment (73). Relevant clinical data from the studies in sets 2-5 are presented in **Table 1**.

**Table 1:** Results of studies on the efficacy of rhGH plus GnRHa on adult height in children with idiopathic short stature.

Design	Uncontrolled studies		Short combo vs no treatment		Combo vs rhGH alone		Adding GnRHa to rhGH treatment	
Ref	(68)	(69)	(70)	(58)	(71)	(72)	(73)	
Diagnosis	ISS	ISS	ISS	ISS+SGA (RCT)	ISS	ISS (RCTdiscont^1^)	ISS prepub	ISS pub
Duration								
rhGH, y	2.3	2	2.5	3	4.6	2.4	7.8 vs 7.0M, 5.9 vs 5.6F	4.2 vs 3.1M, 3.7 vs 2.9F
GnRHa, y	2.3	2	2.5	3	4.6	2.4	2.0M, 1.8F	2.0M, 1.8F
Controls	–	–	Hist, No R/	No R/	Hist, rhGH 4.9y	rhGH, 2.4y	rhGH	rhGH
N, sex	10F	25M, 12F	10 vs 10 3M, 7F	17 vs 15 11M, 21F	12 vs 12 F	19 vs 23M 26 vs 20F	12 vs 62M 19 vs 33F	8 vs 28M 19 vs 11F
Age, y	11.6	13.8M 12.6F	11.8 vs 11.4	11.6 vs 11.8	10.2 vs 10.7	12.1 vs 12.1	8.3 vs 9.4M 9.1 vs 9.0F	12.9 vs 14.3M 11.3 vs 12.7F
HSDS0	-2.7	-2.8M -3.1F	-2.4 vs -2.3	-2.4 vs -2.5	–	-2.5 vs -2.5	-2.4 vs -2.6M -2.7 vs -2.7F	-2.3 vs -2.7M -2.1 vs -2.7F
ΔPAH	3.0 (1y) cm		0.7 vs -0.6 cm	9.3 vs 1.2 cm	10.5 vs 7.9 cm			
AH	-2.8 SDS	-1.8M -0.3F	151.7 vs 150.3 cm	-2.0 vs-2.3 SDS	156.3 vs 146.3 cm	-1.9 vs -1.8 SDS^1^	-0.5 vs -0.8M -0.7 vs -0.7F SDS	-0.5 vs -0.7M -1.1 vs -1.1F
AH-HSDS0	-0.1	2.0M 2.7F		0.5 vs 0.3	–		1.9 vs 1.8M 2.0 vs 1.9F	1.8 vs 2.0M 1.6 vs 1.1F
AH-PAH, cm	1.4	5.3M 12.8F	1.0 vs -1.5	4.4 vs -0.5	10.0 vs 6.1		8.4 vs 6.3M 11.9 vs 12.7F	7.6 vs 4.7M 9.5 vs 7.2F
AH-TH	-3.0 cm	-0.1 SDS M 0.5 SDS F	-10.0 vs -9.6 cm	-6.0 vs -11.2 cm	3.6 vs -4.1 cm		0.6 vs 0.3M 0.2 vs 0.1F SDS	0.1 vs 0.1M 0.3 vs -0.0F SDS
Total pubertal height gain, cm							34.4 vs 26.8M 27.5 vs 18.7F	35.8 vs 29.0M 25.7 vs 21.9F

^1^Trial was abrupted after 2.4 years, thus severe loss to follow-up. NAH was reported on a small sample only.

AH, adult height; Combo, combination; discont, discontinued; F, females; HSDS0, height SDS at start of treatment; hist, historical; ISS, idiopathic short stature; M, males; PAH, predicted adult height; R/, treatment; RCT, randomized controlled trial; SGA, small for gestational age; TH, target height; vs, versus; y, year.

The two uncontrolled studies in set 2 (LoE 4) presented contradictory results. The Italian study (68) showed no positive effect, while the authors of the Chinese study (69) concluded that GnRHa/rhGH therapy can effectively improve AH SDS up to TH SDS.

Similarly, the results of the two studies in set 3 were contradictory. The small prospective Venezuelan study compared the growth response to rhGH plus GnRHa with that of untreated historical controls (LoE 3) and reported no difference (70). In contrast, the RCT of our group in short children born with a low or normal birth size (58) (LoE 1) showed a mean positive effect on AH (5 cm), with a clear difference between sexes: mean (SD) AH-TH was -3.3 (5.9) cm in the treated group versus -12.0 (5.3) cm in untreated controls (p<0.05) in girls, compared with -11.8 (6.5) versus -9.7 (6.9) cm in boys (NS). It is noteworthy that of the PAH increase of 9.3 cm between start and discontinuation of treatment, almost 50% was “lost” between the end of medication and AH. In this study, no long-term negative or positive psychosocial consequences were observed (74), bone mineral density (BMD) did not change significantly, and the effect appeared similar for ISS and SGA (58).

In set 4, the effect of rhGH plus GnRHa for a relatively long period (4.6 years) was compared with that of rhGH-treated matched controls in girls (71). The result was impressive (AH 156.3 ± 5.9 vs. 146.3 ± 5.0 cm, so 10 cm difference). The large RCT comparing the combination treatment with rhGH alone (72) was unfortunately aborted at the request of the French regulatory authorities (LoE 2). NAH was only reported on 35 of the included 91 children, which did not show a difference between treatment regimens. Bone fractures occurred more frequently in the GH plus leuprorelin group than in the GH alone group (seven versus 3, respectively), but no bone fractures were reported during the safety follow-up period.

Finally (set 5), the effect of adding GnRHa in puberty was investigated in a retrospective analysis on 58 out of 192 children who either started rhGH treatment before (n=31) or during puberty (n=27) (73). The authors concluded that combined rhGH/GnRHa therapy increased AH outcome, and that this effect was more pronounced in the prepubertal group and in girls. The mean effect on total pubertal growth was 8-9 cm (LoE 3).

Though strictly speaking short, adopted girls should not be labeled ISS, I also mention in this paragraph two studies on adopted girls showing an estimated mean height gain of 3-4 cm on rhGH plus GnRHa versus GnRHa alone (LoE 1) (75, 76).

The general picture arising from these studies is that in countries where rhGH can be prescribed for children with ISS, clinicians can consider adding a GnRHa if puberty starts when height SDS is still below the reference range at onset of puberty, particularly in girls (58, 71, 73). A longer duration of GnRHa co-medication than 3 years may have a larger effect on AH (71), but its potentially negative psychosocial consequences should be considered as well.

#### 2.2.4 Haploinsufficiency of *SHOX*, *NPR2*, or *ACAN*

In children with haploinsufficiency of *SHOX*, *NPR2* or *ACAN*, the prepubertal growth rate is relatively well-preserved but followed by a compromised pubertal growth spurt due to premature growth plate fusion, so that height SDS decreases by age (77–79). *SHOX* haploinsufficiency is a registered indication for rhGH since 2006 (80), but this does not apply to the two other genetic syndromes. The few anecdotal reports on the administration of GnRHas as co-treatment to rhGH in children with these genetic defects are reviewed in the next paragraphs.

##### 2.2.4.1 SHOX Haploinsufficiency

In a retrospective analysis of 10 children with *SHOX* haploinsufficiency (77), five patients were followed without treatment, and five were treated with rhGH (50 µg/kg.d) plus GnRHa. Mean AH SDS minus height SDS at first evaluation was significantly different between treated (+0.6) versus nontreated (-1.2) patients (77) (LoE 4), but obviously the relative contribution of the two components of this combined therapy is unknown. In a Japanese paper two girls with *SHOX* haploinsufficiency were treated with rhGH and a few years of GnRHa, but the reported data do not allow for assessing the contribution of GnRHa co-treatment (81) (LoE 4).

##### 2.2.4.2 NPR2 Haploinsufficiency

In a recent paper the data of 21 patients with heterozygous *NPR2* variants who were treated with rhGH were summarized. In three of them a GnRHa was added and in one case letrozole (82). The first reported case (83) was treated with a GnRHa for 1.5 years from 13.3 years onward and with rhGH (33 µg/kg.d) for 3.3 years from the age of 13.8 years. Height SDS increased from -2.8 to -2.5 SDS and AH was 158 cm, similar to the heights of his affected father and grandfather, suggesting no beneficial effect (LoE 4).

In the same paper (83), the results were reported of treatment of rhGH (50 µg/kg.d) plus GnRHa in a 12.8 year old boy. Puberty had started at 12.1 years, when he was 131 cm and had a PAH of 160 cm. After 2.7 years of treatment, GnRHa was discontinued. He reached an AH of 164 cm, suggestive for an AH gain of 4 cm (LoE 4).

Growth data of the third case (84), showed that treatment with rhGH for 11.6 years and an unreported duration of GnRHa led to a height SDS increase from -3.8 to -3.1 SDS (82) (LoE 4). Taken together, rhGH plus GnRHa appears to have a modest AH-augmenting effect, but the respective roles of rhGH and GnRHa cannot be assessed in this condition.

##### 2.2.4.3 ACAN Haploinsufficiency

Approximately 50% of children with *ACAN* haploinsufficiency present with an accelerated BA (85) and mean height SDS decreases by age (79), so clinicians have been tempted to investigate the potential role of rhGH plus GnRHa to increase AH. In a large international patient series, five children received rhGH plus GnRHa, which indeed appeared to halt skeletal maturation (79) (LoE 4). In a recent report on 6 novel cases with heterozygous *ACAN* variants, the authors reviewed previously published cases who were treated with rhGH with (n=11) and without (n=10) a GnRHa or an AI (86) (LoE 4). Unfortunately, no separate analysis was made for GnRHs and AIs. No statistically significant differences were found in terms of AH, but the presumably wide interindividual differences in age at start of medication and other relevant clinical variables make it difficult to interpret these findings. Specifically in patients with *ACAN* haploinsufficiency, accelerated epiphyseal fusion during pubertal years remains a worrisome clinical problem and studies investigating add-on GnRHa or AI therapy in these patients should be a priority.

#### 2.2.5 Central Precocious Puberty Associated With a Persistently Low Predicted Adult Height

While in most cases of CPP treatment with a GnRHa leads to an increase of PAH and a normal AH (10), there are patients with CPP in whom PAH remains low on GnRHa alone, usually associated with a very low height velocity. The addition of rhGH to GnRHa treatment in 10 girls for 2-3 years led to an AH gain of 7.9 cm in comparison to 1.6 cm with GnRHa alone in matched controls (87) (LoE 3). Longer follow-up in a larger cohort showed a mean additional AH gain of 6 cm (88) (LoE 3). Of note, rhGH has not been registered for this indication by any of the drug regulatory agencies.

An observational study on rhGH plus GnRHa showed that this treatment was associated with a height gain of 5.4 cm in 18 girls with CPP, compared to 3.0 cm in 62 girls receiving the same treatment but with normal onset of puberty (42) (LoE 4).

A meta-analysis of controlled studies in CPP with severely decreased growth velocity during GnRHa therapy (89) reported an increased AH in patients with GnRHa plus rhGH versus GnRHa alone [+2.8 cm in four clinical controlled trials (CCTs) and +4.3 cm in one RCT], as well as greater AH-TH (+3.9 cm in the CCTs and +4.0 cm in the RCT) and greater AH-PAH (+3.5 cm in the CCTs and +3.9 cm in the RCT). Patients with a low growth velocity or no improvement in PAH during GnRHa benefitted most from the combination therapy (89) (LoE 2). A systematic review and meta-analysis reported an increase of PAH of 6.5 cm in CPP when treated with rhGH plus GnRHa, especially in those starting treatment before 10 years old, or with treatment lasting more than 12 months (90). Compared to GnRHa alone, the combined treatment showed a 3.7 cm higher PAH (LoE2). AH was not reported.

In a large retrospective analysis on girls with CPP or early and rapidly progressive puberty (91), the effect on AH of GnRHa plus rhGH treatment, GnRHa alone, or no treatment were compared. Compared to no treatment, GnRHa alone yielded an AH gain of 1.5 cm and when combined with rhGH an additional 1.5 cm was gained. Compared to TH, GnRHa alone showed an increase of 2.0 cm and combined with rhGH a total increase of 4.0 cm, while controls reached their average TH. An AH-PAH of at least +5 cm was reached in 60% of controls, 70% of GnRHa and 75% of those with combination therapy, especially those with a more advanced BA and low PAH. Lower percentages were seen when the TH-AH endpoint was used (10%, 25% and 45%, respectively) (LoE 3).

#### 2.2.6 Congenital Adrenal Hyperplasia

In general, growth of children treated for CAH stays within the population range if glucocorticoid dosage is minimized (to avoid iatrogenic Cushing’s syndrome), mineralocorticoids are properly dosed and supplemental sodium is given to infants (92). The Endocrine Society Clinical Practice Guideline recommended against routine use of experimental therapies to promote growth and delay puberty (92). However, several retrospective studies showed that mean AH of children treated for CAH is approximately 1 SD below the population mean, probably due to a combination of the condition itself and its treatment (93).

Several groups have tried to increase AH by adding rhGH plus GnRHa (both off-label for this indication) to the conventional treatment with hydrocortisone and fludrocortisone acetate. In a study comparing 2 years of rhGH alone or rhGH plus GnRHa co-treatment versus controls without such treatment, mean PAH increased by 11 cm in both experimental groups combined and closely approximated TH, while PAH did not change in controls. There was no difference between the groups treated with rhGH alone and those treated with the combination of rhGH with GnRHa (94) (LoE 3).

In another study, rhGH plus GnRHa therapy was given for 4.2-4.4 years and results were compared with matched controls. In the treatment group, AH SDS was 1 SD greater than both the initial PAH SDS and AH SDS of the untreated group (95) (LoE 3). Six years later, in a nonrandomized prospective study, the same group reported 34 patients that were predicted to be more than 2 SD below TH or the population mean at around 8 years old, treated with either rhGH alone (n=7) or combined with GnRHa in case of precocious or early puberty (n=27). AH was 9.2 ± 6.7 cm higher than initial PAH in males, and 10.5 ± 3.7 cm in females (96) (LoE 3). There were no differences between the two treatment options, although the rhGH alone group started treatment at a later age. In subjects with poor adrenal control (all males), the gain was only 4.0 ± 3.0 cm.

In a retrospective analysis (97), 13 patients were treated with rhGH plus GnRHa. rhGH was given for 1.0-6.3 years and GnRHa for 2.1.-6.2 years. On average, an increase of 2 SD in height SDS for BA was noted in the first years after treatment, which remained stable until NAH was reached (LoE 3). In another study (98), 32 CAH patients with CPP were treated with GnRHa (n=11), GnRHa plus letrozole (n=11), or no additional treatment (n=10). Compared to no additional treatment, only the GnRHa plus letrozole group had a higher NAH (-1.3 vs -2.5 SDS) (LoE 3). A Chinese retrospective study compared the effect of two regimens: rhGH plus GnRHa and rhGH plus GnRHa plus letrozole (for an average period of 25 months). PAH increased by 9 cm versus 12 cm, respectively (99) (LoE 3).

From the available data I conclude that in the subset of CAH patients with a low PAH at pubertal onset, adding rhGH alone or in combination with a GnRHa may increase AH.

#### 2.2.7 Hypothyroidism

In a case-report two patients with severe acquired juvenile hypothyroidism presenting with compromised PAH were treated with rhGH plus GnRHa in addition to LT4 (100). In the first patient, a 13 year-old girl, PAH decreased to 144 cm after one year of LT4 treatment. rhGH plus GnRHa for one year slowed BA progression, and led to an AH of 155 cm (LoE 4). In the second patient, a 14 year-old boy, a 2 year treatment with rhGH plus GnRHa was initiated in addition to LT4 leading to improvement of growth velocity (10.6 cm/yr) while slowing bone age progression, resulting in an AH equal to TH, an increase of 10 cm compared with PAH after one year of LT4 treatment (LoE 4). When one compares these observations with the study on the effect of the addition of GnRHa alone (36) (paragraph 2.1.1), the addition of rhGH appears crucial for a substantial height gain, but obviously observations in two patients are insufficient for a reliable assessment.

### 2.3 Safety of GnRHas in Childhood and Adolescence

According to the GnRHa consensus meeting (10), “GnRHas are generally well tolerated in children and adolescents. Systemic complaints such as headaches or hot flashes occur occasionally but are usually short-term and do not interfere with therapy. Local adverse events occur in 10-15% patients and necessitate a change in agent when persistent, because they can result in sterile abscesses in a fraction of the patients. Although exceedingly rare, anaphylaxis has been described”. The International Consortium gave a very elaborate description of possible adverse events, but basically the conclusion was the same: “Adverse effects of GnRHa therapy are rare, and the associations of most reported adverse events with the GnRHa molecule itself are unclear. Decades of experience have shown that GnRHa treatment is both safe and efficacious” (11). In a few studies negative effects on bone acquisition have been reported (27, 45, 58), but these appear transient (47).

The main downside of GnRHa treatment is that it brings the early- or mid-pubertal adolescent back to a prepubertal state, not suitable to his or her age, which may lead to psychosocial issues and differences in behavior and interests compared with peers.

## 3 The Effect of Aromatase Inhibitors

While GnRHas can be considered as having a “semi-physiologic” effect (by bringing the body back to a prepubertal state), AIs are clearly pharmacological agents. They are registered for the treatment of estrogen-dependent breast cancer, and aimed at decreasing the exposure to estrogens as much as possible, primarily by inhibiting the intracellular conversion of androgens to estrogens, but also by decreasing circulating estrogen concentrations. At present, there are three so-called third generation AI compounds registered: letrozole (2.5 mg o.d.), anastrozole (1 mg o.d.) and exemestane (25 mg o.d.). The effect of letrozole on aromatase inhibition is stronger compared to that of anastrozole [88% vs 85% tissue aromatase blockade in postmenopausal women (101)], with mean residual estradiol concentrations of 10.1% for anastrozole and 5.9% for letrozole (102), consistent with observations in boys with ISS (103). Exemestane is a nonsteroidal aromatase inhibitor, and covalent binding of the drug to the active site of the enzyme irreversibly inhibits aromatase action, in contrast to the two non-steroidal AIs which form a reversible bond with the enzyme [reviewed in (5)].

AIs are not registered for any pediatric indication and as far as I know no basic pharmacological studies have been performed in children or adolescents. Therefore, no information is available on the optimal dosages for the three compounds if they would be used in childhood and adolescence. In fact, in pediatric studies the same dosage has been used as for adults, which probably leads to maximum suppression of aromatase. The use of AIs has been investigated in four groups of conditions: hyperestrogenism, hyperandrogenism, pubertal gynecomastia, and short stature and/or delayed puberty (5).

### 3.1 Use of Aromatase Inhibitors in Hyperestrogenism, Hyperandrogenism, or Pubertal Gynecomastia

Four rare disorders characterized by hyperestrogenism (aromatase excess syndrome, Peutz-Jeghers syndrome, McCune-Albright syndrome, and functional follicular ovarian cysts) are logical indications for AIs. For details I refer to a previous review (5) and a recent paper on Peutz-Jeghers syndrome (104).

Regarding hyperandrogenism, the efficacy of AIs in testotoxicosis, also known as familial male-limited precocious puberty, is well established (5). The positive effect of long-term treatment with the combination of an antiandrogen, aromatase inhibitor and GnRH analogue was recently confirmed (105, 106) (LoE 2).

As mentioned in paragraph 2.2.6, the conventional treatment of children with 21-hydroxylase deficiency with a combination of hydrocortisone and fludrocortisone acetate may in some children lead to a low AH. A lower dosage of corticosteroid treatment in combination with a first generation AI and an androgen antagonist appeared efficacious (107), but became outdated by the arrival of second and third generation AIs.

The addition of letrozole to CAH patients with concomitant CPP who received a GnRHa led to a significantly higher NAH compared with the no intervention group (98) (LoE 3). In a recent case report the effect of 9 years of exemestane, combined with 4 years of GnRHa treatment because of early central puberty, was described in a patient with markedly advanced BA (108) (LoE 4). At start of exemestane the PAH was -4.6 SDS and at its discontinuation NAH was -0.8 SDS. In another study, already mentioned in paragraph 2.2.6, the addition of letrozole to rhGH plus GnRHa had an additional effect of 3 cm (99) (LoE 4).

Also patients with an 11 β-hydroxylase deficiency can end up short, particularly if the diagnosis is made during later childhood. The first report on 11 years of administration of letrozole without rhGH in a boy with 11β-hydroxylase deficiency demonstrated a 35 cm increase in AH over PAH (109) (LoE 4). This was combined with a GnRH analogue for 2.5 years due to central activation of puberty. However, significant adverse events were reported in this patient: back pain and vertebral changes were noted from the age of 15 years onwards, as well as impaired sperm motility and subnormal morphology. Two case reports showed that the addition of rhGH and an AI to glucocorticoid replacement was highly efficacious (LoE 4). The first case (110) was treated with rhGH and letrozole resulting in a higher AH as compared with PAH. The second case (111), treated with rhGH plus anastrozole, reached an AH of 11.5 cm above TH. In these patients no side effects were noted.

While initially AI treatment appeared to be a rational approach in boys with pubertal gynecomastia, the results have been disappointing (5). In a recent clinical practice guideline the use of selective estrogen receptor modulators, aromatase inhibitors, or non-aromatizable androgens was not recommended for this self-limiting condition (112).

### 3.2 Use of Aromatase Inhibitors in Boys With Short Stature and/or Delayed Puberty

Third generation AIs have been used as a potential agent to enhance AH, particularly letrozole (2.5 mg o.d.) and anastrozole (1 mg o.d.). The motivation to initiate such studies was the observation that males with a pathogenic variant of the genes encoding the estrogen receptor or aromatase show a substantial BA retardation while their stature in childhood and adolescence was normal, resulting in increased AH (14, 15, 113–116).

Regarding the use of AIs in children, there is one systematic review by the Cochrane group (117), which concluded that the “available evidence suggested that aromatase inhibitors improved short-term growth outcomes”, but that “there was no evidence to support an increase in AH, based on limited data, with only one of four trials publishing AH data under non-randomized conditions” (LoE 2).

In most reports and reviews on AIs, the authors apparently assumed that the effects of letrozole and anastrozole are similar regarding growth and skeletal maturation. In contrast, I believe that there is circumstantial evidence that the effects differ to some extent, which may well be related to the different degree of blocking aromatase. The biphasic dose-response relationship of estrogens and growth discussed in paragraph 1 would suggest that a total blockade of estrogen exposure may have a negative effect on growth, which could serve as an argument in favor of anastrozole above letrozole.

Unfortunately, there is little information about a direct comparison between the efficacy and safety of both compounds in childhood. There is only one study in which the effect was studied on growth and BA in short pubertal males. First year height velocities were similar, but PAH increased more in the anastrozole group (118) (LoE 3). The study in which either letrozole or anastrozole was administered to boys with ISS (103) was not powered to detect differences between the effect of these drugs on growth and skeletal maturation. Similarly, there is no direct comparative information about adverse events in adolescents of both compounds, but the published data seem to suggest that adverse events are seen more frequently on letrozole treatment than on anastrozole.

In the following paragraphs I summarize the outcome of clinical studies in children/adolescents at risk of a low AH, as a follow-up to several previous reviews (1, 3, 5, 119–122).

#### 3.2.1 Idiopathic Short Stature, Growth Hormone Deficiency, and Constitutional Delay of Growth and Puberty

Several studies have been performed with AIs in boys with ISS, constitutional delay of growth and puberty (CDGP) (which I consider equivalent to ISS with delayed onset of puberty) or GHD (5). Auxological data from several studies are presented in **Tables 2**, **3**. Given the possible differences in efficacy and safety between letrozole and anastrozole, the results will be discussed in separate paragraphs for studies on letrozole, anastrozole and studies in which both were used.

**Table 2:** Effect of letrozole (Let) or anastrozole (Ana) on growth in boys with idiopathic short stature (ISS).

Country, Diagnosis (duration)	Finland, ISS (2 yrs)		US, ISS, 1 yr		US, ISS, 2 yrs		
Reference	(123, 124)		(118)		(103)*		
Medication	Let	Placebo	Let	Ana	Let/Ana	GH	Let/Ana+GH
N	10	10	17	22	25	25	26
**At start**							
Age, yrs	11.5 (1.8)	10.9 (1.8)	14.1 (1.3)	14.1 (1.4)	14.2 (0.2	14.1 (0.2)	14.0 (0.2)
Hgt, cm	129.7 (7.9)	127.5 (7.5)	148.7 (6.2)	149.3 (6.7)	145.7 (1.1)	144.2 (1.4)	144.5 (1.3)
Hgt SDS	-2.4 (0.3)	-2.5 (0.4)			-2.2 (0.1)	-2.4 (0.1)	-2.3 (0.1)
HV, cm/yr			7.1 (3.0)	6.0 (3.5)			
TH, cm			175.3 (4.5)	173.8 (8.7)	171.8 (0.8)	170.1 (1.3)	171.6 (0.9)
BA, yrs	9.2 (2.6)	8.7 (1.9)	13.3 (0.7)	13.4 (0.8)	12.8 (0.3)	12.9 (0.3)	12.7 (0.2)
PAH, cm	167.6 (7.9)	166.9 (3.9)	166.4 (4.5)	165.7 (5.2)			
Testic vol, ml	1.5 (1.4)	1.0 (0.6)	8.3 (3.2)	7.7 (3.5)			
Tanner G	8/2/0/0/0	10/0/0/0/0	0/8/8/1/0	0/9/9/4/0	2-3	2-3	2-3
T, nmol/L	1.4 (1.9)	0.4 (0.4)			7.1 (1.3)	8.5 (1.4)	7.7 (1.3)
**At stop**							
Age			15.2 (1.3)	15.2 (1.5)			
Hgt, cm			156.4 (5.1)	157.6 (6.7)			
Hgt SDS					-1.73 (0.1)	-1.43 (0.1)	-1.25 (0.1)
HV, cm/yr			7.2 (2.1)	7.2 (1.8)			
Δ hgt,cm					14. (0.8)	17.1 (0.9)	18.9 (0.8)
BA, yrs	10.2 (2.9)	10.8 (1.5)	14.2 (0.8)	14.2 (0.9)			
ΔBA, yrs	1.24#	2.05#			2.1 (0.3)	2.5 (0.1)	1.9 (0.2)
PAH, cm	174.0 (8.3)	167.4 (4.3)	167.7 (5.6)	169.9 (6.3)			
HSDSBA					-1.06 (0.1)	-1.11 (0.2)	-0.41 (01)
ΔPAH, cm	6.4 (2.2)	0.5 (4.4)	1.4 (4.4)	4.4 (3.5)			
Testic vol, ml			14.3 (3.3)	14.0 (2.7)			
Tanner G	5/0/1/1/3	3/3/2/2/0	0/0/4/12/1	0/1/7/10/3	4-5		
**At (near-)AH**							
Age	23.3 (4.0)	21.7 (3.1)			17.4 (0.2)		
Hgt, cm	164.8 (4.0)	163.7 (3.7)			164.1 (1.6)	164.8 (1.6)	166.9 (1.5)
NAH-TH					-7.8 (1.6)	-5.3 (1.3)	-4.5 (1.4)
ΔHgt, cm					18.2 (1.6)	20.6 (1.5)	22.5 (1.4)
ΔHgt 3yrs					23.8 (2.3)	26.7 (2.0)	30.7 (1.1)
ΔHgt 2yrs					14.7 (1.5)	17.8 (1.6)	19.9 (1.4)
Hgt SDS	-2.6 (0.7)	-2.7 (0.7)			-1.4 (0.1)	-1.4 (0.2)	-1.0 (0.1)
BA, yrs	18.5 (0.7)	18.7 (0.7)			15.3 (0.1)		
Testic vol, ml	12.8 (3.0)	12.2 (3.5)					

*Data from Mauras et al. are expressed as mean (SE). Data from other papers are expressed as mean (SD).

^#^Derived from Hero et al, 2005 on 16 and 14 patients, respectively.

AH, adult height; BA, bone age; G, genital stage according to Tanner; GH, growth hormone; Hgt, height; HV, height velocity; HSDSBA, height SDS for bone age; ISS, idiopathic short stature; NAH, near-adult height; PAH, predicted adult height; SDS, standard deviation score; T, testosterone; testic, testicular; TH, target height; vol, volume; yrs, years.

**Table 3:** Effect of anastrozole (Ana) or letrozole (Let) in boys with growth hormone deficiency (GHD) or idiopathic short stature (ISS).

Country, Dg	US, GHD		France, ISS			US, GHD versus ISS	
Reference	(125)		(126)			(127)	
Medication	Ana+GH	Placebo+GH	Ana+GH	GH	Controls	GH+[Ana/Let]	GH+[Ana/Let]
N	26	26	12	12	17	115	27
**At start**							
Age, yrs	13.8 (0.3)	14.2 (0.2)	15.2 (0.8)	15.2 (1.1)	15.1 (0.8)	14.7 (1.9)	13.8 (1.7)
Hgt, cm	149.7 (1.6)	151.6 (13)	155.0 (4)	156.3 (2.9)	156.1(3.5)		
Hgt SDS	-1.4 (0.2)	-1.5 (0.2)	-1.7 (0.7)	-1.7 (1)	-1.7 (0.8)	-1.0 (0.9)	-1.0 (0.8)
TH, cm	169.8 (1.6)	173.1 (1.2)	-1.15	-1.2	-1.15		
BA, yrs	13.7 (0.2)	13.4 (0.2)	14.5 (0.8)	14.6 (0.6)	14.6 (0.7)	13.5 (2.4)	13.5 (1.0)
BA/CA						0.97 (0.10)	0.99 (0.10)
PAH, cm			157.9 (3.8)	158.2 (2.9)			
PAH SDS			-2.9 (0.6)	-2.84 (0.5)			
Testic vol, ml			22.2 (5)	22.4 (8)	22 (5)		
Tanner G	2-4	2-4					
T, nmol/L	7.5 (1.2)	5.6 (1.0)	5.6 (0.9)	5.5 (0.9)	5.5 (0.8)		
**At 1-2 yrs**	**At 2 yrs**		**At ≈1 yr (NAH)**			**At 1 yr**	
N	? (total 41)		12	12		72	19
Duration GH	2 yrs	2 yrs	19 (5.9)mo	11.5 (5)mo			
Age, yrs			16.8 (0.7)	16.2 (1.1)			
Hgt, cm	162.9 (1.4)	166.6 (1.4)	168.4 (2.6)	164.2(5.6)	160.1(2.8)		
Hgt SDS 0						-0.92 (0.9)	-0.87 (0.9)
Hgt SDS			-1.1 (0.9)	-1.8 (0.9)		-0.62 (1.0)	-0.69 (0.8)
Δ hgt,cm			12.7 (5.6)	7.8 (5)			
BA, yrs	15.4 (0.2)	16.0 (0.2)					
BA/CA						0.93 (0.1)	0.96 (0.1)
ΔBA, yrs	1.8 (0.1)	2.7 (0.1)					
ΔHSDSBA	0.5 (0.1)	0.0 (0.1)					
ΔPAH, cm	4.5 (1.2)	1.1 (1.1)					
NAH-PAH, cm			10.5 (5.2)	5.9 (4.5)			
T, nmol/L	21.8 (2.5)	19.8 (2.0)					
**At 2-3 yrs**	**At 3 yrs**					**At 2 yrs**	
N	? (total 28)					27	9
Hgt, cm	165.8 (1.3)	167.8 (1.8)					
Hgt SDS 0						-1.00 (1.0)	-0.85 (0.9)
Hgt SDS						-0.40 (1.2)	-0.65 (0.5)
BA, yrs	15.9 (0.3)	17.2 (0.3)					
ΔBA, yrs	2.5 (0.2)	4.1 (0.1)					
ΔHSDSBA	0.8 (0.2)	-0.1 (0.2)					
BA/CA						0.95 (0.1)	0.96 (0.06)
ΔPAH, cm	6.7 (1.4)	1.0 (1.1)					
T, nmol/L	17.7 (2.2)	11.7 (1.1)					

BA, bone age; CA, chronological age; G, genital stage according to Tanner; Hgt, height; Hgt SDS 0, height SDS at baseline; HSDSBA, height SDS for bone age; mo, months; NAH, near-adult height; PAH, predicted adult height; SDS, standard deviation score; T, testosterone; testic, testicular; TH, target height; vol, volume; yrs, years.

##### 3.2.1.1 Letrozole

The first RCT on the effect of letrozole in boys with CDGP (128, 129) appeared to lead to a small gain of NAH (LoE 1), but as discussed previously (5) there are several issues which make this claim uncertain. The most important issue is potential selection bias at start of treatment and in the final analysis. Further, results obtained with a combination treatment (letrozole plus testosterone) cannot be extrapolated to letrozole alone. In addition, no AH data were reported for the untreated boys, and the attained AH might be considerably higher than the NAH, since the range of bone ages at follow-up was quite wide (15.8–18.0 years).

The same Finnish group also performed an RCT on the effect of a 2 year course of letrozole vs. placebo in peripubertal boys with ISS (prepubertal in 90%) (**Table 2**). At discontinuation of medication there was a 5.9 cm increase of PAH on letrozole (123), but the authors have to be praised for performing a study on attained AH. This showed that there was no statistically significant difference in AH (124) (LoE 1). Of note, in both groups, the attained AH was 3 cm lower than PAH at start of treatment (124). Regarding the study design, in hindsight, it would have been more logical to start letrozole in early- or midpuberty than before puberty. Minor vertebral deformities occurred in boys who were prepubertal at start (130), but this ameliorated with time (124). The results of this study serve as a warning against overestimating the accuracy of PAH calculations after a therapeutic course with a puberty modulator.

In an Iranian study, 91 boys with CDGP were randomly allocated to letrozole, placebo or oxandrolone for 2 years (131). The results showed several questionable issues (5) and unfortunately we have been unable to obtain clarifications from the authors (LoE 4). In another Iranian study (132) the effect of one year on letrozole versus no treatment in small groups of 6 boys was reported. The authors concluded that letrozole treatment was associated with an AH of 1.9 cm above PAH, in contrast to no increase in controls (0.1 cm). However, the letrozole group was investigated at a considerably older age than the controls (23.4 versus 19.9 years) (LoE 3).

In a retrospective chart review of 21 boys with predicted short stature and/or rapidly advancing BA, due to many different diagnoses, 19 received letrozole, one subject anastrozole, and one subject initially anastrozole for 1 year followed by a switch to letrozole. No increase of PAH was observed, regardless of Tanner stage (2) (LoE 4).

Finally, a recent Finnish randomized controlled phase 3 trial tested whether letrozole for 6 months might be a feasible alternative treatment to low-dose testosterone for boys with CDGP (133). Thus, this study was not aimed at investigating whether letrozole would result in a taller AH. In the letrozole group serum concentrations of LH, FSH, testosterone and inhibin-B as well as testicular volume were higher than in the testosterone group, but height velocity was slightly lower (LoE 1). The safety profile of both regimens was satisfactory, but the authors warned that “the risks and benefits of manipulating the reproductive axis during early puberty should be weighed carefully” (133).

A case report of a GH deficient boy who received letrozole for 17 month in addition to rhGH suggested a positive effect on PAH, but unfortunately no data on attained AH were reported (134) (LoE 4). Another case report on the effect of 5 years of letrozole in a 14.5-year-old boy with ISS demonstrated that AH surpassed the pre-treatment PAH by 15 cm (135) (LoE 4).

##### 3.2.1.2 Anastrozole

In an RCT from the USA, 52 adolescent males with GH deficiency treated with rhGH were randomized to cotreatment with anastrozole or placebo daily for up to 36 months. Anastrozole cotreatment was associated with slower bone maturation and higher PAH while maintaining normal pubertal progression after 2–3 yr (125) (LoE 1) (**Table 3**). Unfortunately, no AH data could be collected. Furthermore, the interpretation of the results is complicated because of a potential selection bias, since the number of patients who could be analyzed decreased from the original 52 to 41 and 28 completing 2 and 3 years, respectively, without information on the distribution per group.

An interesting approach was taken by a French group (126), which explored the effect on AH of rhGH plus anastrozole, compared with rhGH alone and historical untreated controls, by the end of puberty in boys with ISS (**Table 3**). In this small study, rhGH plus anastrozole, despite being started at such late stage of puberty, seemed to allow boys with ISS to reach a greater AH than rhGH alone (LoE 2).

##### 3.2.1.3 Letrozole or Anastrozole

As mentioned previously, first year data from a direct comparison of anastrozole and letrozole in children with ISS confirmed that letrozole is more potent in hormonal manipulation, but suggested that PAH increased more in the anastrozole group (118) (LoE 3). Unfortunately, long-term results of this study have not been reported, but even if these would be available, the lack of an untreated control group would have hampered the interpretation.

An RCT in 76 boys with ISS consisted of three arms: 1) AI (letrozole or anastrozole); 2) rhGH, and; 3) AI plus rhGH (103). The authors concluded that AI plus rhGH for 24–36 months increased height potential in pubertal boys with ISS more than rhGH or AI alone (LoE 1), but unfortunately no data have been reported on attained AH. The effect was considerably greater if patients were treated for at least 36 months, but this could only be investigated in the 19 out of 54 boys with a residual height potential at 24 months who chose to continue treatment, resulting in potentially selective loss to follow up. As mentioned earlier, the study was not powered to investigate differences between letrozole and anastrozole in terms of efficacy and safety. The total QoL scores increased significantly at 24 months in the rhGH and AI plus rhGH group, but QoL derived from the children’s reports did not increase in the AI group, while it increased in all groups in the parents’ reports. Increases in QoL scores were associated with increases in height SDS (136).

A retrospective assessment of the effect of anastrozole or letrozole with or without rhGH treatment for a mean period of 2.1 years was performed in 96 adolescent boys with, as stated in the report, an “idiopathic decrease in PAH when compared with TH” (137). In contrast, the baseline data showed that mean pre-treatment PAH was close to mean TH (range -3.4 to +2.4 cm around TH) and mean height SDS ranged between -1.0 and 0.0. The normal stature of these boys and low number of patients who reached NAH (n=22), as well as the retrospective design, preclude firm conclusions (LoE 4).

In an observational study from the United States (127) on boys with GHD or ISS, with a similar loss to follow up and risk of selection bias as mentioned for the RCTs, the authors concluded that the addition of an AI may augment growth potential as indicated by continued height SDS increase with decreased BA/chronological age (BA/CA) ratio (**Table 3**) (LoE 4).

A recent study from China reported on a comparison of the effect of rhGH alone or combined with letrozole or anastrozole for one year or more (138). After intervention, there were significant differences in ΔBA/ΔCA, ΔHeight SDS for BA and ΔPAH between the rhGH plus AI group and the rhGH group (LoE 3). However, multiple adverse events were reported (see later).

#### 3.2.2 Haploinsufficiency of *SHOX*, *NPR2*, and *ACAN*

There are only a few anecdotal reported data on the combination of rhGH plus AI in children with these genetic variants. One child with a heterozygous pathogenic *NPR2* variant was treated with rhGH in a dosage of 50 µg/kg.d and letrozole (2.5 mg/d) since he was 13 years old (83). His height SDS remained stable and his AH prediction based on his bone age improved from 156 cm to 167.4 cm during therapy (LoE 4).

In one case of the large cohort of patients with *ACAN* haploinsufficiency (79), the effect of letrozole treatment for one year was reported, resulting in arrested bone maturation (LoE 4). In a recent Chinese study (86), the effect of GnRHa or an AI in addition to rhGH was compared with rhGH alone, but no separate analysis of these two interventions were presented, as discussed earlier (LoE 4).

#### 3.2.3 Hypothyroidism

There is one case report on the effect of anastrozole as cotreatment to LT4 treatment in a boy who was diagnosed at 12 years of age with a height of -3.5 SDS (139). After 2 years of LT4, anastrozole was added for 1.5 years because of rapid bone maturation, resulting in a PAH of 11 cm below TH. BA advancement slowed and the patient’s NAH was 2.4 cm taller than PAH at start of anastrozole (LoE 4).

#### 3.2.4 Chronic Kidney Disease

In a case report, a one year treatment with anastrozole after renal transplantation appeared to have a positive effect on growth and the authors suggested that this strategy should be considered in children who present with significant short stature close to puberty when time for other therapeutic options is limited (140) (LoE 4).

### 3.3 The effect of Aromatase Inhibitors in Girls

For the four rare disorders characterized by hyperestrogenism (aromatase excess syndrome, Peutz-Jeghers syndrome, McCune-Albright syndrome, and functional follicular ovarian cysts) AIs in girls have been used with modest results (5). Regarding hyperandrogenism, the combination of letrozole and a GnRHa has been applied to 7 girls with CAH and CPP, with apparently a good effect on AH (98) (LoE 4).

Most clinicians are hesitant to use AIs in girls in order to increase AH, and in the most recent review AIs were considered “contraindicated in girls with short stature due to concerns about precipitating ovarian cysts with risk of torsion” (3), based on the occurrence of ovarian torsion in patients with congenital aromatase deficiency. Still, a prospective phase 2a study was performed in 40 girls consecutively referred for early puberty (onset 7.5–9 years) with a PAH <−2 or >1.5 SD lower than their TH, which compared GnRHa plus anastrozole (n=20) with GnRHa alone (n=20) for 2 years or until the age of 10 years (141). In the group receiving the combination treatment, PAH SDS gain was almost double of that observed in the group on GnRHa alone by 12 and 18 months, and reached the maximum of +1.2 SDS (7.5 cm) vs +0.3 SDS (1.9 cm) after 2 years, respectively (LoE 2).

### 3.4 Safety of Aromatase Inhibitors in Childhood and Adolescence

The use of AIs in male children and adolescents has been associated with several adverse events, probably associated with a decrease of circulating and intracellular estrogens and an increase of circulating androgens due to increased LH secretion, particularly on letrozole.

Estrogen receptors and aromatase activity are ubiquitously present throughout the body, suggesting that estrogen signaling is crucial for many tissues. The main potential adverse events associated with estrogen deficiency are changes in BMD, bone turnover, lipid metabolism, insulin sensitivity, and cognitive performance (1, 3). In fact, adults with estrogen deficiency due to hypogonadism or aromatase deficiency do present with a low BMD. Although in the RCTs no change of BMD was reported (130), a temporary decrease of BMD was noted by Krebs et al. (135).

In the study on the effect of letrozole in boys with ISS, most of whom were prepubertal boys at start of treatment, vertebral anomalies were noted (142). A similar observation was made in a boy treated for 11 years from 4 years of age onward (109). Serum HDL slightly decreased and insulin sensitivity slightly increased (143). No significant adverse effect on cognitive performance was found (1). Increased erythropoiesis was noted in several studies (2, 144–146). A theoretical risk of AIs is that letrozole-induced gonadotropin secretion and ensuing high concentrations of intratesticular testosterone might affect development of seminiferous epithelium (133). In fact, abnormalities of sperm (impaired motility and subnormal morphology) were observed in the boy treated with letrozole for 11 years (109), but not in an RCT on the effect of anastrozole (147).

Multiple adverse events were noted during follow-up of the 151 patients treated with rhGH plus letrozole or anastrozole, including elevated uric acid, decreased HDL, severe acne, excitement, hyperactivity and irritability, a fracture, mild renal dysfunction, inactivity, drowsiness, memory loss and performance decline, mildly abnormal liver function, and granulocytopenia (138). The percentages of knee pain (11-12%) and impaired fasting glucose (1-2%) were similar to those in patients on rhGH alone. In another report (2), the severity of acne and hematocrit significantly increased in boys who started treatment in Tanner IV-V. In girls no adverse events were observed (141).

Although no head-to-head comparison is possible, my impression is that adverse events of letrozole are more frequent than of anastrozole.

## 4 Discussion and Conclusion

In general, the scientific evidence for the efficacy of GnRHa or AIs in the treatment of various growth disorders is suboptimal, because of the scarcity of randomized controlled trials up to AH and conflicting reports. **Table 4** shows my subjective impression of the efficacy of GnRHa or AI alone, or various combinations, in particular with rhGH.

**Table 4:** Apparent efficacy of different treatment regimens manipulating skeletal maturation for increasing adult height.

	GnRHa alone	GnRHa + rhGH	GnRHa + AI	AI alone	AI + rhGH
CPP	++	+^1^	±		
Hypothyroidism^2^	±	±			
Laron syndrome	±				
GHD		++^3^			±
SGA		+^3^			
ISS	±	+ in girls, ± in boys		±	±
*SHOX*, *NPR2*, or *ACAN* haploinsufficiency		±			±
CAH		+^3^	± ^3^		±
CDGP				±	±

^1^In case of severely decreased growth velocity during GnRHa therapy.

^2^In case of severe longstanding hypothyroidism and bone age delay.

^3^In case of precocious puberty or when entering puberty at a low height SDS.

++: Treatment considered effective in significantly increasing adult height, based on several good quality studies.

+: Some treatment effect on adult height with reasonable certainty.

+/-: Uncertain treatment effect due to conflicting or limited evidence.

CAH, congenital adrenal hyperplasia; CDGP, constitutional delay of growth and puberty; CPP, central precocious puberty; GHD, growth hormone deficiency; ISS, idiopathic short stature; SGA, small for gestational age.

The efficacy of a GnRHa alone is proven for children with CPP, but less certain for any other condition. The available clinical evidence appears sufficient to conclude that in rhGH-treated children with GHD or SGA and in rhGH-treated girls with ISS who develop CPP or who are short at pubertal onset, AH can be increased by adding a GnRHa for 2-3 years. Anecdotal reports suggest that a larger effect can be reached with a longer treatment period, for example in girls with ISS and relatively early puberty. However, the potential adverse consequences in terms of bone health and psychosocial development have to be considered.

A combination of rhGH plus GnRHa has also been used for children with several conditions, if PAH SDS was low at the onset of puberty. The effect appears similar among conditions, and close to a gain of 1 SD (6-7 cm). However, the number of RCTs is low, much of the evidence is derived from uncontrolled studies and case reports, for most conditions rhGH treatment is not registered, and GnRHas can only be prescribed off-label except for CPP. Still, I believe that the similarity between the results in children with the various conditions, particularly children with CPP on GnRHa treatment with a low height velocity and PAH and those in children with CAH with a low PAH, render it plausible that growth stimulation by rhGH in combination with low estrogen exposure by a GnRHa provides more time for growth by inhibiting maturation of the epiphyseal growth plates and increases AH. These observations are also in line with the observations on experiments of nature (continued growth and tall AH in disorders where circulating estrogens are low). GnRHa treatment is usually tolerated well, and a modest decrease of BMD appears to be compensated after discontinuation of treatment.

Theoretically, one would expect a similar effect of AIs on growth and skeletal maturation as of GnRHas, but the lack of reported data on AH make it difficult to estimate the extent of the effect. AIs do have a therapeutic value in a few very rare disorders, but their value in growth disorders, either alone or in combination with rhGH, is still uncertain. In patients with GHD or ISS, co-medication with anastrozole (103, 125) may have a similar effect as co-medication with a GnRHa (45), but the lack of AH data precludes a firm conclusion. Long-term follow-up of the boys with ISS treated with letrozole have shown that a statistically significant increase of PAH does not always translate to an increased AH (124). Comparing anastrozole and letrozole co-medication, the results tend to be slightly superior for anastrozole, but this has to be confirmed by an RCT comparing the two compounds directly.

AI treatment leads to increased plasma testosterone, which is considered a psychological advantage for adolescent males but a potential disadvantage for females. Therefore, the use of AIs in adolescence has virtually be confined to boys. The preliminary data on the use of anastrozole in short girls (141) challenge this hypothesis, but this observation needs confirmation.

Regarding safety, GnRHas are considered safe (11), but there is more uncertainty about the safety of AIs, particularly if treatment is given for a long period, and possibly more for letrozole than for anastrozole.

## Author Contributions

The author confirms being the sole contributor of this work and has approved it for publication.

## Conflict of Interest

The author declares that the research was conducted in the absence of any commercial or financial relationships that could be construed as a potential conflict of interest.

## Publisher’s Note

All claims expressed in this article are solely those of the authors and do not necessarily represent those of their affiliated organizations, or those of the publisher, the editors and the reviewers. Any product that may be evaluated in this article, or claim that may be made by its manufacturer, is not guaranteed or endorsed by the publisher.

## Abbreviations

AH, adult height; AIs, aromatase inhibitors; BA, bone age; BMC, bone mineral content; BMD, bone mineral density; BMI, body mass index; CA, chronological age; CAH, congenital adrenal hyperplasia; CCTs, clinical controlled trials; CDGP, Constitutional delay of growth and puberty; CPP, central precocious puberty; GH, growth hormone; GHD, growth hormone deficiency; GnRH, Gonadotropin Releasing Hormone (GnRH); GnRHa, Gonadotropin Releasing Hormone (GnRH) analogue; HRQoL, Health-related Quality of Life; ISS, idiopathic short stature; NAH, near-adult height; PAH, predicted adult height; LT4, Levothyroxine; RCT, randomized controlled trial; rhGH, recombinant human growth hormone; SDS, standard deviation score; SGA, small for gestational age, also used for a short child born small for gestational age; TH, target height.
